# Identifying and Classifying Pollution Hotspots to Guide Watershed Management in a Large Multiuse Watershed

**DOI:** 10.3390/ijerph14030260

**Published:** 2017-03-03

**Authors:** Fangli Su, David Kaplan, Lifeng Li, Haifu Li, Fei Song, Haisheng Liu

**Affiliations:** 1 College of Water Conversation, Shenyang Agricultural University, No. 120 Dongling Road, Shenyang 110866, China; xiyue_li@163.com (L.L.); syaulhf@126.com (H.L.); sfsongfei@163.com (F.S.); liuhaisheng929@syau.edu.cn (H.L.); 2 Liaoning Shuangtai Estuary Wetland Ecosystem Research Station, Nanjingzi Village, Dongguo Town, Panshan County, Panjin City 124112, Liaoning Province, China; 3 Engineering School of Sustainable Infrastructure and Environment, Department of Environmental Engineering Sciences, University of Florida, 6 Phelps Lab, Gainesville, FL 32611-6350, USA; dkaplan@ufl.edu

**Keywords:** classification, water quality, watershed management, cluster analysis, Dahuofang reservoir, point source pollution, non-point source pollution

## Abstract

In many locations around the globe, large reservoir sustainability is threatened by land use change and direct pollution loading from the upstream watershed. However, the size and complexity of upstream basins makes the planning and implementation of watershed-scale pollution management a challenge. In this study, we established an evaluation system based on 17 factors, representing the potential point and non-point source pollutants and the environmental carrying capacity which are likely to affect the water quality in the Dahuofang Reservoir and watershed in northeastern China. We used entropy methods to rank 118 subwatersheds by their potential pollution threat and clustered subwatersheds according to the potential pollution type. Combining ranking and clustering analyses allowed us to suggest specific areas for prioritized watershed management (in particular, two subwatersheds with the greatest pollution potential) and to recommend the conservation of current practices in other less vulnerable locations (91 small watersheds with low pollution potential). Finally, we identified the factors most likely to influence the water quality of each of the 118 subwatersheds and suggested adaptive control measures for each location. These results provide a scientific basis for improving the watershed management and sustainability of the Dahuofang reservoir and a framework for identifying threats and prioritizing the management of watersheds of large reservoirs around the world.

## 1. Introduction

Large-sized reservoirs are the main water sources for irrigation, reservoir cultivation, hydroelectric power, and drinking, in major inland populations. The water quality and quantity of the reservoir is determined by many factors in the upstream watershed, such as the non-point source pollution, point source pollution, land use, population density, domestic wastewater discharges, herbicide concentrations, bacterial and viral contamination, and so on. Managing the upstream watershed is an essential way to control the water of the reservoir. While the upstream watershed is always complex, formed by hundreds of small watersheds, it is becoming popular to manage the whole watershed on a small watershed scale.

### 1.1. Water Quality Impacting Factors in Watersheds

It has been generally recognized that the quality of surface water is affected by the watershed. An example of this is Lake Tahoe, where urbanization around the lake has greatly increased. This has led to an increased nutrient flux into the lake, resulting in increasing algae production and rapidly declining water clarity over the past 50 years [1]. Another study shows that the significant difference between the nitrogen input and nitrogen output loads is associated with the irrigation system, which is fed by the N-rich Po River, suggesting that this basin metabolizes part of the nitrogen excess produced upstream [2].

Additionally, studies have shown that non-point source pollution has become the largest driver of reservoir water quality in the 21st century [3], particularly agricultural pollutants, such as nitrogen (N), phosphorus (P), and sediment (NPS). When NPSs are released into aquatic environments, they cause a range of problems, such as NPS pollution load. For instance, the agricultural land was identified as the primary pollutant source in the Three Gorges Reservoir Region, China [4]. An increasing nitrogen concentration in upstream surface-waters could significantly affect the water quality, and land cover and fertilization practices were the major determinants [5]. Non-point source pollution often flows into downstream rivers and reservoirs through soil erosion [6].

The close relationship between the land use type and the water quality of watersheds has also been widely accepted. A study of the Chaohu Lake Basin indicated that there was a significantly negative correlation between forest land, grassland, and water pollution. Moreover, the construction built-up area had negative impacts on the water quality, while the influence of the cultivated land on the water quality was very complex [7,8,9].

Many factors can affect water quality. In Buyukcekmece Lake, the third largest water source in Turkey, a remarkable portion of the pollution in the watershed has been attributed to domestic wastewater discharges, and agricultural areas that occupy more than half of the watershed have also adversely affected the water quality [10]. Herbicide concentrations in the lake water were thought to reflect agricultural cultivation of the watershed [11]. Moreover, viruses can affect water the quality. Human and bovine viruses in the watershed, affected by emergencies, hydrologic variability, and seasonal variability, can infiltrate the surface water [12]. Therefore, it is necessary to establish a system of comprehensive impact indicators for evaluating water quality.

### 1.2. Assessing and Managing Methods of Water Quality Impacting Factors

A growing number of statistical methods are being used to evaluate sources of water pollution from the watershed. Principal components analysis (PCA) was used to identify sources of emerging organic contaminants in Southeastern Minnesota and was considered a useful way to broadly categorize the sources of new and previously uncharacterized emerging contaminants [13]. The methods were also used to investigate a temporal assessment of the land use/cover of the Omerli Watershed and the water quality changes in the reservoir [14]. Regression Tree Analysis is examined as a tool to evaluate hydrological and land use factors that affect the nitrate and chloride stream concentrations during low-flow conditions, which significantly enhanced the model’s accuracy over multiple linear regressions, increasing the nitrate R^2^ values and chloride R^2^ values [15]. Principal component analysis and stepwise multiple regression analysis were used in the river watersheds of China, to examine the relationship between landscape characteristics and water quality, at both spatial and temporal scales [16,17].

Aerial photography of the watershed can be analyzed and classified by GIS software, to monitor changes in land cover and the subsequent environmental responses to water quality, especially over a long period of time [18]. A sophisticated multivariate analysis of covariance (ANCOVA) provided estimates of both seasonal and overall load reductions in a paired watershed, to evaluate changes in P loading of the Cannonsville Reservoir a drinking water supply for New York City. This indicated that changes in farm management practices and physical infrastructure clearly produced decreases in event P losses, measurable at the small watershed scale [19]. Recently, a decision support system (DSS), consisting of the Maryland Phosphorus Index (PI), diagnosis expert system (ES), prescription ES, and a nonpoint-source pollution model, the Ground Water Loading Effects of Agricultural Management Systems (GLEAMS), was developed and applied to an agricultural watershed in southern Sweden. It showed that DSS may enable people to select a proper BMP implementation strategy and to realize the beneficial effect of BMPs on a long-term basis [20].

### 1.3. Managing on a Small Watershed Scale

Some findings suggest that current pollution controlling strategies are not sufficient for protecting water quality. These strategies should consider the influence on small upland streams and protect small watersheds located downstream [21]. A small watershed is defined as an independent and completely natural catchment area, shaped by a main gully and bounded by the watershed and the outlet section, with an area between 300 to 5000 hm^2^. The small watershed is a unit of independent soil erosion, animal husbandry, industry, and economy. It is easy to govern a separately enclosed area on a small watershed scale [22]. It is becoming increasingly popular to solve natural resource issues and related economic problems on a small watershed scale, as demonstrated by much of the research conducted at the Hubbard Brook Experimental Forest (HBEF) in New Hampshire [23].

Another example is the Natural Resources Conservation Service (NRCS) in North Dakota. The projects under the Small Watershed Program have studied how to map flood hazard areas, solve local flooding problems, evaluate potential greenbelts along streams, develop guidelines for erosion control and runoff management, help farmers control erosion in high priority watersheds, and improve water quality to water bodies and ground water. North Dakota has four watershed projects that are in various stages of implementation [24].

The advantages of managing a watershed on a small watershed scale are obvious. It can provide an accurate measurement of nutrient input and output (erosion), therefore establishing the relationship between the smaller ecosystem and the larger biosphere cycles. It can also provide a means of studying the interrelationships between the biology and the hydrologic cycle, various nutrient cycles, and energy flow in a single system. The effect of various land-management practices or environmental pollutants on nutrient cycling in natural systems can be tested in a small watershed. Therefore, the management of the whole watershed can be realized by dividing the whole watershed into small watershed structures.

However, some problems still exist. For example, the watersheds of a reservoir often occupy hundreds of small watersheds, which is difficult to comprehensively govern under limited financial and human resource conditions. Therefore, the key areas and pivotal factors affecting the water quality should be controlled first, while other areas can be postponed. That is to say, different parts of the watershed should be managed, depending on their impact on the water quality. However, due to a lack of research, this type of implementation is restricted. In addition, land use types are always diversified in reservoir watersheds, and this diversification affects water quality via many factors. Existing proposals are still deficient, and target controlling measures should be studied.

This research is the first to be conducted in the Dahuofang Reservoir Upstream Watershed of China. Recently, due to the increased population growth and rapid economic development, the resource exploitation rate is increasing, along with intensifying point source and non-point source pollution, decreasing biodiversity, a declining forest coverage rate, and increasing soil erosion. The water safety of this increasingly fragile ecological environment is thus being threatened. This research will establish a water quality evaluation system and value pollution index for every small watershed upstream, and then classify these into different groups, based on the amount of control required. The results will provide scientific guidance for the protection of water source areas. Different controlling water quality projects will be implemented, depending on the pollution source.

## 2. Materials and Methods

### 2.1. Study Area

**Figure 1:**
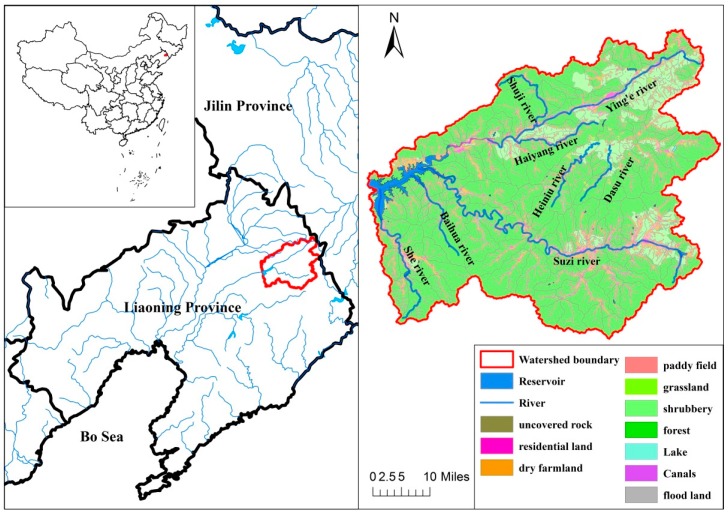
Subwatersheds and major land uses in the Dahuofang watershed. The 11,000 ha reservoir is shown in blue in the eastern portion of the basin.

The 8761 km^2^ Dahuofang watershed is located in the Liaoning Province in northeastern China (Figure 1). The region has a temperate, continental, monsoon climate, with an average annual temperature of 3.5 °C to 7.8 °C, and average precipitation of 752 to 1100 mm. The elevation of the watershed ranges from 1126 m in the mountains, to 97 m in the lower plains. The main soil types are brown soil, dark brown soil, meadow soil, meadow dark brown soil, cinnamon soil, and paddy soil. The watershed remains largely vegetated (>70% coverage) by deciduous forests and scrublands, with only about 8% of the area having been converted for agricultural use (Figure 1). The region is rich in mineral resources, such as gold and iron, and is also home to a number of mineral quarries and brick factories. The human population in the watershed is 624,400 and has grown steadily in the past 30 years, with the majority of people living in the four medium-sized cities of Shenyang, Fushun, Benxi, and Anshan (Figure 1).

The land use types of the Dahuofang Reservoir watershed changed significantly in the ten years between 2004 and 2013, which is determined by the area of different biological specimens on the land. The orchard area declined in the beginning period and began to rise after 2010; the forest area sustained a slight increase; the grassland area continued to decrease; the urban land area continued to rise steadily; the wasteland area decreased; and the dry agricultural and paddy fields remained unchanged over the 10 years. The change in land use types had various outcomes. For example, the reduction of the orchard area reduced the amount of fertilizer and pesticides introduced into the environment.

Urbanization occupied abundant grasslands and wastelands, and developed into construction, which also increased the soil erosion. Although the area of soil erosion (the total area of water erosion, wind erosion, and freeze-thaw erosion) has decreased from 152,080 ha to 115,709 ha in the past 10 years, the soil erosion intensity increased rapidly. The area of strong and very strong erosion consistently increased until 2010, when it decreased slightly. Severe erosion appeared in 2010 and then kept increasing until 2013, accompanied by a period of rapid urbanization. Therefore, we can conclude that the increase in erosion intensity appeared with the development of construction. This phenomenon also increased the non-point source pollution and water degradation of the reservoir.

The Dahuofang Reservoir, built in 1958, serves as a key water control project in the region, and provides flood control, irrigation supply, power generation, aquaculture, and other important services. It is also the drinking water source for seven large cities in the Liaoning Province. It has a volume of 21.87 hundred million m^3^, with an installed capacity of 32,000 kilowatt. The average depth is 12 m and the deepest point is 36 m. In sum, the Dahuofang Reservoir provides 83% of all water resources in the Liaoning Province. The water quantity and quality of the reservoir is thus a critical determinant of human health and economic and social development in the Liaoning province.

The watershed of the Dahuofang Reservoir is 11,000 ha and occupies 47.4% of the Hunhe River watershed, with a 543,700 ha catchment area. There are 118 small watersheds in this watershed. The small watershed is identified by the Water and Soil Conservation Bureau of the Liaoning Province (WSCBLN, 2012) [25].

### 2.2. Evaluation Index Development

Indicators that may affect the water quality were investigated and collected as extensively as possible. The area of paddy fields, dry land, and soil erosion parameters were interpreted from remote sensing films. The usage amounts of compound fertilizer, nitrogen fertilizer, phosphorus fertilizer, pesticide, organic fertilizer-N, and organic fertilizer-P were provided by the local Bureau of Agriculture and Forestry. The area of farmland and the number of livestock (such as pig, sheep, and poultry) were provided by the local Bureau of Animal Husbandry. The quantity of the population, industry outfall, and RDW (rural domestic waste), were collected from the local Environmental Protection Agency. The identification and boundary of 118 small watersheds were cited from the Forth Remote-sense Investigation of the Liaoning province (WSCBLN, 2012) [25]. From the 17 relevant and available data sets, we identified four broad categories of potential impact: (1) non-point source pollution; (2) environmental bearing; (3) point source pollution; and (4) soil erosion (Table 1).

### 2.3. The Weight of Evaluation Indexes

The weight of evaluation indexes, which is divided into subjective and objective methods, is essential to the evaluation results in this multi-index comprehensive system analysis. The subjective methods reflected the preference and willingness of the experts [26], such as the Delphi Method and AHP (Analytic Hierarchy Process) [27], while the objective methods determined the weight of each evaluation index by calculating the exceeding rate, reflecting the contribution of ratios of data to the evaluation results [28]. One hundred respondents completed questionnaires as part of the subjective methodology, whose specialties covered the environment, soil, plants, and hydrology etc.

In addition, the entropy method is a new method for determining the weights of evaluation indexes. Entropy is a concept of thermodynamics, introduced to information theory by C. E. Shannon, and is widely applied in engineering, technology, and social economics [29,30,31]. It can determine the weight of an index by calculating the valid information from data [32], and is objective and dependable [33]. When the discrepancy of one index is large among samples, its entropy value is small, and this means that the index can provide more valid information, as well as being heavily weighted. The opposite also holds true. When the value of an index is the same among the samples, the entropy value reaches a maximum value, which means that the index cannot provide any useful information for decision-making. This index can therefore be removed from the evaluation index system.

Supposing there are *m* evaluation objects and evaluation indexes, the original data matrix is formed as:

(1) $$X={\left(x_{ij}\right)}_{m\times n}=\left(\begin{array}{ccc} x_{11} & \cdots & x_{1n}\\ \vdots & \ddots & \vdots \\ x_{m1} & \cdots & x_{mn} \end{array}\right)$$

1. Standardization of index data matrix

The standardization of the matrix is:

(2) $$R={\left(r_{ij}\right)}_{m\times n}$$

In the above equation, the standardization value of the *jth* index in the *ith* small watershed is $r_{ij}\in \left[0,1\right]$. This can be represented as:

(3) $$r_{ij}=\frac{x_{ij}-x_{j\min}}{x_{j\max}-x_{j\min}}\left(i=1,2,\cdots ,n,j=1,2,\cdots ,m\right)$$

2. Defining the entropy

The entropy of the *jth* index is defined as follows:

(4) $$e_{j}=-k\sum_{i=1}^{n}\left(P_{ij}\times \ln\;P_{ij}\right)$$

In the above equation:

(5) $$P_{ij}=r_{ij}/\sum_{i=1}^{n}r_{ij},k=1/\ln\;n,\;\mathrm{when}\;P_{ij}=0,P_{ij}\times \ln\;P_{ij}=0$$

3. Defining the entropy weight

The entropy weight of the *jth* index is defined as follows:

(6) $$w_{j}=\frac{1-e_{j}}{m-\sum_{j=1}^{m}e_{j}}$$

In the above equation:

(7) $$\sum_{j=1}^{m}w_{j}=1(0\leq w_{j}\leq 1)$$

4. The composite score of each small watershed is calculated by the synthetical index method, based on data of the weight.
  (8) $$P(W)=\sum_{i=1}^{n}{X_{i}}_{j}\times W_{j}$$

Interpretation: *P(W)* is the composite score of the small watershed. *W_j_* is the weight of the *jth* index. *X_ij_* is the standardization value of each index. The water pollution is serious when the comprehensive score of the small watershed is higher, indicating the need for urgent management.

## 3. Results

### 3.1. The Weight of Factors Affecting Water Quality

The factors affecting water quality have different units. In order to reduce the error of this reason, the indicator data were normalized for each watershed using Formula (2). The degree of every factor affecting the water quality was valued by calculating the weight of each parameter, based on the entropy method (as described in Section 2.3). It included 118 small watersheds upstream of the Dahuofang Reservoir.

As shown in Table 2, the most serious factor affecting the water quality is non-point resource pollution, which accounts for 41% of all indicators. This means that, as an agricultural watershed, the fertilizer and pesticide use by the Dahuofang Reservoir should be strictly controlled. The second most serious factor is the environmental bearing with a value of 35%, the third most important is point source pollution with a value of 19%, and the least serious factor is soil erosion, at 5%.

An analysis of each parameter shows that industry outfall, as a point source pollution factor, has the highest index weight, with a value of 0.1207. This indicates that the effect of industry emissions on the water quality is larger than any other parameter. Industry outfall is closely followed by sheep breeding, with a weight of 0.1 under the environmental bearing factor.

The survey results show that there are 347.68 km^2^ of dry fields and 81.52 km^2^ of paddy fields in the whole watershed (WSCBLN, 2012). About 750 kg of basal fertilizer, mostly compound fertilizer, is used per dry field hectare, and 600 kg of fertilizer is used per paddy hectare. In addition, pesticides, mostly in the form of herbicides, are also used. About 13.5 kg of pesticide per hectare is used in dry fields and 7.5 kg per hectare is used in paddy fields. Statistical results show that about 51,340.65 tons of fertilizer and 571.57 tons of pesticide are put into the whole upstream watershed per year. The extensive use of chemical fertilizers and pesticides has led to soil compaction and a decrease in the water conservation capacity of the soil. Moreover, herbicide runs off into the channels and rivers, and eventually flows into the Dahuofang reservoir, where it causes water pollution.

### 3.2. Small Watershed Evaluation Results

**Figure 2:**
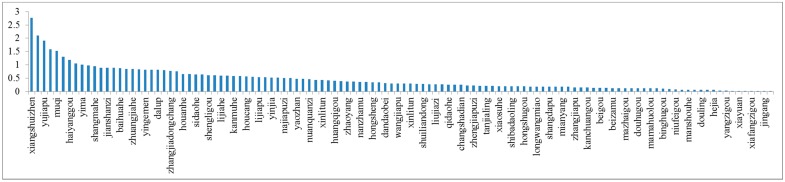
Comprehensive score of small watershed.

The index weight of each small watershed indicates its effect on the water quality. The scores from Figure 2 are the summation of the whole parameters, calculated via Formula (8). The higher the score, the greater the impact on the water quality. As shown in Figure 2, the weight of Xiangshuizhen is the highest, with a value of 2.76, which indicates it has the largest impact on the water quality of the Dahuofang Reservoir; it also means that it is the site which is most in need of management. A small watershed with a lower score needs to continue to maintain its current status, such as Fulaigou.

**Figure 3:**
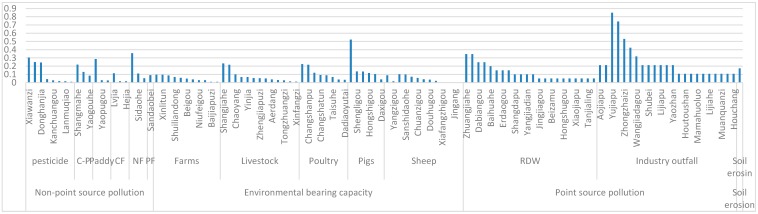
The classification figure of the small watersheds. Note: C-P means phosphorus in organic fertilizer; CF means compound fertilizer, PF means phosphorus fertilizer; NF means nitrogen fertilizer.

There are hundreds of small watersheds in the reservoir upstream watershed, which played different roles in determining the water quality. Limited by the capital and manpower, choosing the most serious small watersheds and managing them with scientific and reasonable measures, is feasible. This can be achieved by examining the standardized value of each small watershed and the weight of each index, from Figure 1 and Figure 3. The small watershed score is calculated according to Formula (8). For example, the highest parameter of Xiangshuizhen is the amount of industry outfall, with a score of 0.7426. This means that industry emission is the key factor affecting the water quality. Thus, the most important measure for this watershed is controlling industry emission by reducing heavily polluting enterprises, and improving the pollution purification and sewage discharge standard. The results are shown in Figure 2 and Figure 3.

### 3.3. Small Watershed Classification

**Figure 4:**
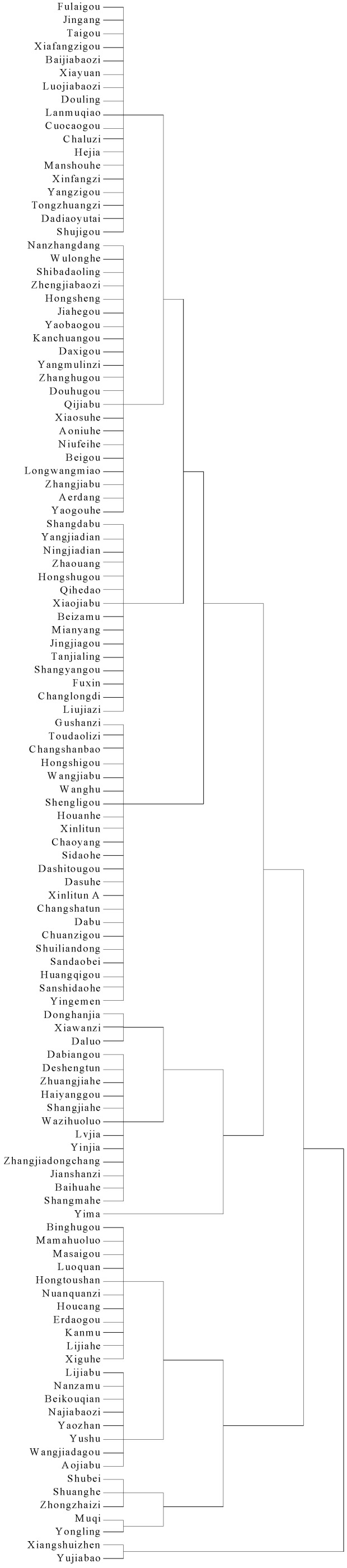
The result of K-means clustering analysis.

**Figure 5:**
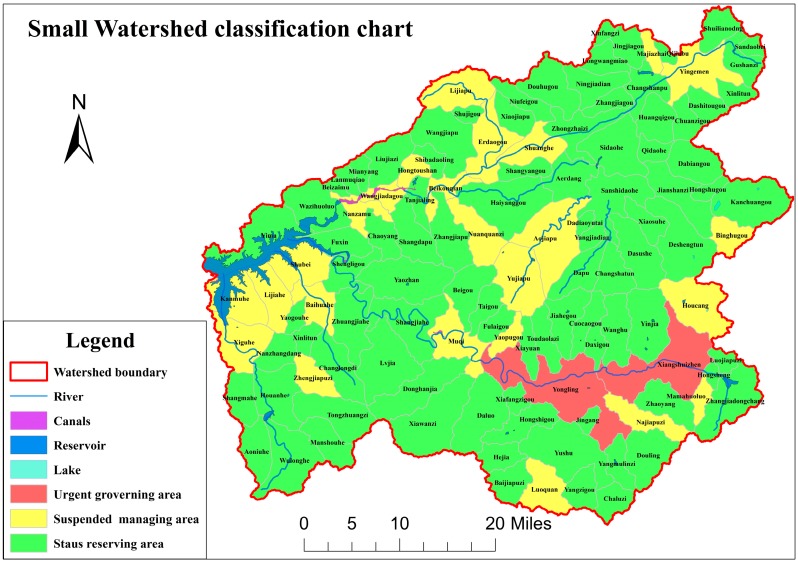
The distribution map of the K-means clustering analysis result in the upstream of the Dahuofang reservoir.

For the purpose of conveniently managing the small watersheds, this study divided the 118 small watersheds into three groups. A K-means clustering analysis method is used, as shown in Figure 4, which is based on the score of non-point source pollution, point source pollution, environment load bearing, and soil erosion in each small watershed. The distribution map is shown in Figure 5.

The classification of 118 small watersheds in the upstream watershed of the Dahuofang Reservoir is as follows:

1. Urgent management area

This area includes two small watersheds, the Xiangshui and Yongling, with areas and scores larger than other watersheds. The indexes of point source pollution and non-point source pollution are higher than the other indexes, in almost all aspects. Therefore, management should focus on controlling fertilizer, pesticide, sewage, and garbage dumping in these small watersheds.

2. Suspended management area

This area includes 25 small watersheds, such as Aojiapu, Lijiapu, and Hongtoushan, among others. The weight of these small watersheds is lower than the first area. The highest indexes focus on non-point source pollution and animal husbandry. The classification result demonstrates that these watersheds have a certain influence on water quality, but that they are not very significant. Therefore, the management measures can be implemented when capital and human resources are all abundant. Furthermore, the amount of animal breeding and industry sewage should be reduced at first.

3. Preserved status area

This area includes 64 small watersheds, such as Aerdang, Aoniuhe, and Yushu, among others, with the lowest weights. Almost all of these watersheds have a large proportion of forest, and the impact of pollution is not significant. The management measures for this classification should focus on prevention and protection against vandalism.

## 4. Discussion

Livestock is an important component of economics, as well as of the factors affecting the water quality of the Dahuofang Reservoir watershed. A reasonable evaluation of the livestock is reliable for the whole watershed. The rational evaluation of livestock and poultry breeding of small watersheds is calculated according to the grassland area. In China, the theoretic carrying capacity (TCC) is used as an indicator to evaluate grassland productivity, which can be calculated as follows [34]:

(9) $$\begin{array}{r} \mathrm{TCC}=(M_{1}\times Y_{1}\times K_{11}\times K_{21}\times K_{31}+M_{2}\times Y_{2}\times K_{12}\times K_{22}\times K_{32}+M_{s-1}\times Y_{m-1}\times K_{1m-1}\\ \times K_{2m-1}\times K_{3m-1}+M_{m}\times Y_{m}\times K_{1m}\times K_{2m}\times K_{3m}÷1825\;\mathrm{kg}\;(\mathrm{SU}) \end{array}$$

*M_m_* is the grassland area (hm^2^); *Y_m_* is the grass yield per unit area (kg/hm^2^); *K*_1*m*_ is the available coefficient of grassland; *K*_2*m*_ is the coefficient of edible forage grass; *K*_3*m*_ is the utilization factor; and SU is the sheep unit (one 40 kg ewe). Chen considered that the maximum carrying capacity is 5.88 million SU of the Altay grazing system, with an area of 34.86 × 104 km^2^ [35]. Calculated according to the above criteria, the theoretical carrying capacity is 92,300 sheep in the study area of 5472.65 km^2^. This is equal to 16.87 sheep units per square kilometer. However, the current number of cattle is 40,148 and sheep is 82,000, which is equivalent to 25.99 SU/km^2^ and exceeds the theoretical load capacity by 54%. The result indicates that the amount of livestock farming was far beyond the scope of the environmental carrying capacity and should be controlled immediately.

Evaluation results from Section 3.2 show that the weight of large livestock, sheep, and poultry, is the highest. It reveals that animal husbandry is one of the main factors of water pollution in the Dahuofang Reservoir upstream watershed. The results are consistent with that calculated by the TCC method. Therefore, although the net income is about $302–$377 per sheep in northern China, this study will not advocate raising sheep by overloading the environmental carrying capacity.

The forest coverage rate reaches 75% in the study area. Because of the forest’s protection, soil erosion is not significant, and the annual erosion was 1,843,418 tons in 2012. According to the formula M = Q/S, M is the erosion modulus, Q is the erosion amount, and S is the area (From: Standards for classification and gradation of soil erosion, Water conservancy industry standard of the People’s Republic of China, SL 190-2007). Soil loss intensity can be calculated as 336.8 t/km·a. On the basis of the Standards (SL 190-2007, China), the average soil erosion of the study area is at a moderate level (M = 200–1500 t/km·a). The results from Section 3.2 also revealed that erosion is not a major cause of water pollution. Therefore, increasing the forest coverage is one effective measure for preventing the pollution of reservoirs.

The traditionally absent livestock farming practices and consequent low use of manure as fertilizer, pose the risk of excess soil mineralization and the progressive loss of denitrification capacity in this area [2]. The animal husbandry industry should be encouraged to maintain a proper amount and extent, in order to maintain the amount of farm manure, rather than the use of chemical fertilizer.

A K-means clustering analysis method is proposed in this study, to divide the 118 small watersheds into three groups. Yi proposed a weighted K-means clustering method based on the blind separation of sparse sources, and achieved considerably accurate results which can be demonstrated by numerical experiments [36]. Matías used K-means clustering and Linear Discriminant analysis for the selection of training and testing sets, and divided 58 derivatives into two clusters. The results show that a molecular descriptor correctly discriminates 100% of the compounds of each cluster [37]. So K-means clustering analysis is a good method for classifying multiple study objects into featured groups.

## 5. Conclusions

The results show that non-point pollution is the foremost factor of the upstream reservoir watershed influencing the water quality. The extensive use of chemical fertilizers and pesticides led to eutrophication.

In order to determine the extent of the impact on the Dahuofang Reservoir, an entropy analysis method was used to calculate the composite score of each small watershed. The results show that the Yongling small watershed makes the greatest impact on the water quality of the reservoir and urgently needs to be managed. The small watershed has an area of 16.84 km^2^, with a population of 27,886, and is about 30 km away from the reservoir. There are many land use types, such as paddy fields, dry land, aquaculture, etc. It is the most important sheep raising base in the upstream of the reservoir, as well as also being one of the key industrial, agricultural, and economic regions. Livestock breeding, especially sheep, should be controlled in this small watershed.

According to the results of K-means clustering analysis, 118 small watersheds in upstream watershed of the Dahuofang Reservoir are divided into three classification districts: urgent management area, suspended management area, and preserved status area. Pollution control measures are proposed, depending on the maximum weight of the small watershed.

## Figures and Tables

**Table 1:** Evaluation factors.

Water quality effecting factors	**Classification**	**Parameters**	**Value**	**Unit**
	Non-point source pollution (A)	Paddy fields (X_1_)	8152.14	hm^2^
		Dry land (X_2_)	34,768.3	hm^2^
		Compound Fertilizer (X_3_)	14,742.64	t
		Nitrogen Fertilizer (X_4_)	18,572.16	t
		Phosphorus Fertilizer (X_5_)	4189.54	t
		Pesticide (X_6_)	571.57	t
		Organic Fertilizer-N (X_7_)	9597.42	t
		Organic Fertilizer-P (X_8_)	4238.89	t
	Environmental bearing (B)	Farm (X_9_)	356	-
		Large Livestock (X_10_)	40,148	-
		Pig (X_11_)	138,051	-
		Sheep (X_12_)	82,000	-
		Poultry (X_13_)	11,703,976	-
		population (X_14_)	368,414	-
	Point source pollution (C)	Industry Outfall (X_15_)	60	-
		RDW (X_16_)	115	-
	Soil erosion (D)	Erosion (X_17_)	1,843,418.01	t

Note: RDW means rural domestic waste.

**Table 2:** The index system and weight of the small watershed classification upstream of the Dahuofang Reservoir.

The Target Layer	The Criterion Layer	The Index Layer	The Index Weight
Small watershed partition	Non-point source pollution (A)	Paddy fields (X_1_)	0.0653
		Dry land (X_2_)	0.0296
		Compound Fertilizer (X_3_)	0.0433
		Nitrogen Fertilizer (X_4_)	0.0588
		Phosphorus Fertilizer (X_5_)	0.0750
		Pesticide (X_6_)	0.0386
		Organic fertilizer-N (X_7_)	0.0358
		Organic fertilizer-P (X_8_)	0.0624
		**Total**	**0.4087**
	Environmental bearing (B)	Farm (X_9_)	0.0443
		Large livestock (X_10_)	0.0477
		Pig (X_11_)	0.0525
		Sheep (X_12_)	0.1013
		Poultry (X_13_)	0.0699
		population (X_14_)	0.0368
		**Total**	**0.3525**
	Point source pollution (C)	Industry outfall (X_15_)	0.1207
		RDW (X_16_)	0.0710
		**Total**	**0.1917**
	Soil erosion (D)	Erosion (X_17_)	0.0463

Note: RDW means rural domestic waste; The index weight of non-point source pollution is 0.4087 which is the total of paddy fields, dry land, compound fertilizer, nitrogen fertilizer, phosphorus fertilizer, pesticide, organic fertilizer-N and organic fertilizer-P. Environmental bearing and point source pollution is 0.3525 and 0.1917 calculated in the same way. Soil erosion calculated directly is 0.0463.
